# Genome-Wide Analyses of Human Respiratory Syncytial Viruses Provide Insights into Evolutionary Dynamics

**DOI:** 10.1093/gbe/evaf093

**Published:** 2025-05-26

**Authors:** Lu-lu Chen, Chu-ci Tong, Yu-xian Zhao, Yan-peng Zheng, Xiang-lei Peng, Yuan-hui Fu, Jin-sheng He, Jie-mei Yu

**Affiliations:** College of Life Sciences and Bioengineering, Beijing Jiaotong University, Beijing 100044, China; College of Life Sciences and Bioengineering, Beijing Jiaotong University, Beijing 100044, China; College of Life Sciences and Bioengineering, Beijing Jiaotong University, Beijing 100044, China; College of Life Sciences and Bioengineering, Beijing Jiaotong University, Beijing 100044, China; College of Life Sciences and Bioengineering, Beijing Jiaotong University, Beijing 100044, China; College of Life Sciences and Bioengineering, Beijing Jiaotong University, Beijing 100044, China; College of Life Sciences and Bioengineering, Beijing Jiaotong University, Beijing 100044, China; College of Life Sciences and Bioengineering, Beijing Jiaotong University, Beijing 100044, China

**Keywords:** human respiratory syncytial virus, genetic diversity, selective pressure, evolutionary pattern, lineage-defining amino acid

## Abstract

Human Respiratory syncytial virus (HRSV) is a leading cause of acute lower respiratory tract infections. It is essential to monitor its genomic characteristics. In this study, we analyzed the variation and evolutionary features of HRSV A and HRSV B using whole-genome data, with a focus on their evolutionary features post-COVID-19. Our findings revealed: (i) the mutation rates of HRSV A genes were generally higher than those of HRSV B genes, with the primary mutation directions for both subtypes being C to T, T to C, G to A, and A to G; (ii) multiple lineages of both subtypes that were prevalent during the pandemic are no longer circulating, likely related to the founder effect caused by non-pharmaceutical interventions; (iii) the lineage-defining amino acids on the neutralizing antigens F and G of the circulating lineages post SARS-CoV-2 pandemic exhibited significant temporal specificity; (iv) HRSV B predominated over A in 2023, and the lineage-defining amino acids of the HRSV B F protein located on or very close to major neutralizing antigenic sites, and several lineage-defining amino acids of the G protein were under strong positive selection. These observations suggested that the HRSV B showed stronger adaptive evolutionary features compared to HRSV A post-pandemic. Combining with the fact that several lineage-defining amino acids are located in the replication-related proteins, we hypothesized a potential model of synergistic evolution mediated by multi-protein mutations in the adaptive evolution of circulating strains. However, the impact of these amino acid changes on the viral properties requires further experimental validation.

**Significance**

This study emphasized the crucial need for continuous monitoring of the evolutionary dynamics of Human Respiratory Syncytial Virus (HRSV), especially after the altered transmission patterns observed during and after the COVID-19 pandemic. As a leading cause of severe respiratory illness in infants, the elderly, and immunocompromised individuals, understanding the genomic diversity and evolution pattern of HRSV is vital for developing effective vaccines and public health strategies. Our analysis highlighted distinct variation patterns between HRSV subtypes A and B post-pandemic, revealing that HRSV B may exhibit significant adaptability to host immune pressure, which may affect future outbreak dynamics. These insights from whole-genome data analysis will enhance our understanding of HRSV evolution, inform ongoing surveillance efforts, and guide the implementation of newly introduced recombinant F protein vaccines, as well as monoclonal antibodies. This research is essential for addressing the ongoing public health challenges posed by HRSV and improving health outcomes in vulnerable populations.

## Introduction

Human respiratory syncytial virus (HRSV) is a significant viral pathogen responsible for acute lower respiratory illnesses (ALRI) in infants, the elderly, and immunocompromised adults. It is the leading cause of pneumonia in children under five in developing countries (Collaborators 2018; Pneumonia Etiology Research for Child Health Study G 2019) and ranks as the second most common pathogen leading to infant mortality due to infection (Graham et al. 2015). Globally, HRSV infections are estimated to result in approximately 33 million cases, 3.2 million hospitalizations, and 59,600 deaths annually among children under 5 years old (Shi et al. 2017). The social and economic burden associated with RSV infections is estimated to be comparable to, or even exceed, that of seasonal influenza (Chiu et al. 2010). Consequently, the genomic features, transmission dynamics, and evolutionary patterns of HRSV have garnered significant attention.

HRSV belongs to the family *Pneumoviridae*, is an enveloped virus, and has a single-stranded negative-sense RNA genome composed of 10 genes that encode 11 separate proteins. Among these proteins, the fusion glycoprotein (F) and the attachment glycoprotein (G) serve as critical neutralizing antigens and are involved in viral attachment (Levine et al. 1987; Feldman et al. 2000; McLellan et al. 2013). The F protein mediates fusion with neighboring cells to form syncytia, exhibits high conservation and strong immunogenicity, and the neutralizing antibodies it induces provide effective cross-protection against viral diseases, making it a primary target antigen for the development of RSV vaccines (Ruckwardt et al. 2019). The G protein exhibits greater variability than the F protein, resulting in significant strain diversity. Although HRSV has a single serotype, it can be categorized into two subtypes, A and B, based on antigenic differences using G protein-specific monoclonal antibodies (Anderson et al. 1985; Mufson et al. 1985). The polymerase L and its cofactors N, P, and M2-1 mediate viral transcription and genome replication (Cao et al. 2021), while M2-2 plays a role in the switch between transcription and replication, highlighting its association with the ribonucleocapsid complex (Bermingham and Collins 1999; Blanchard et al. 2020). The nonstructural proteins NS1 and NS2 primarily facilitate the evasion of the host's innate immune response by interacting with components of different immune signaling pathways (Sedeyn et al. 2019).

Initially, HRSV genotypes were defined based on statistically supported phylogenetic clades derived from the second hypervariable region of the G gene (Peret et al. 1998). While this approach was convenient and cost-effective, recent years have seen proposals for alternative phylogenetic reclassifications using complete genomic data (Chen et al. 2022; Goya et al. 2024). These complete genomic datasets allow for a better understanding of variations across different genes. HRSV subtypes A and B can co-circulate each season, but typically one subtype predominates, with the two subtypes alternating in dominance at 1- to 3-year intervals during epidemics (Bouzas et al. 2016; Song et al. 2023). Prior to the 2020 to 2021 season, HRSV consistently displayed a characteristic epidemic peak, however, COVID-19 pandemic altered this pattern, leading to a seasonality shift and a delayed RSV outbreak with a greater number of infected patients reported in several countries (Weinberger Opek et al. 2021; Pinana et al. 2024). Although our previous study indicated that the evolutionary patterns of RSV did not change during the pandemic (Guo et al. 2023), changes in its transmission dynamics were observed. Therefore, there is a pressing need for more extensive analysis of whole-genome data to support ongoing monitoring of genetic variations in HRSV. This need is particularly urgent in light of the introduction of recombinant F protein vaccines for RSV and the widespread use of the monoclonal antibody Nirsevimab in the United States in 2023. It is unclear how these advancements will affect the mutation patterns of the virus, highlighting the necessity for continued genetic surveillance.

To gain insights into the evolutionary characteristics and variation patterns of HRSV at the genome-wide level, this study analyzed HRSV whole-genome data from public databases. We focused on the evolutionary features of each gene, with particular attention to changes in lineage-defining amino acids on the F and G proteins of the currently circulating strains post-pandemic. The results indicated that the variation patterns of HRSV A and B genes differed, specifically, the circulating strains of HRSV A exhibited relatively conserved antigenicity, while HRSV B demonstrated strong adaptability to immune pressure from the host. The findings provide important reference data for the epidemiological study of RSV, the development of effective vaccines, and the investigation of specific therapies.

## Results

### Sequence Information

**Fig. 1.**
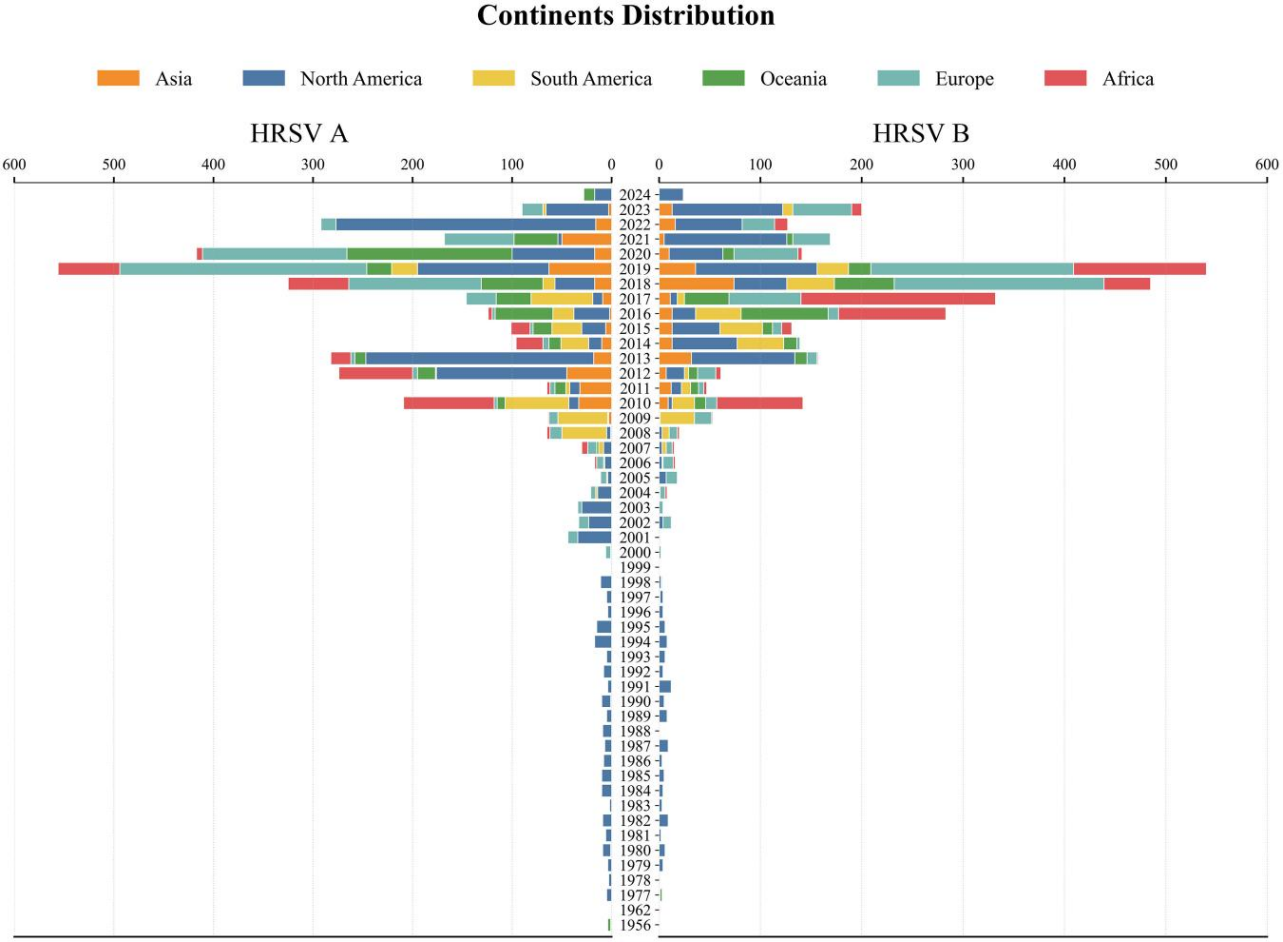
Collection time and spatial distribution of HRSV sequences in this study. The number of sequences was relatively low prior to 2010, with a significant increase observed thereafter. The highest number of sequences originated from North America.

We obtained a total of 8,958 nearly complete genome sequences from the database. After filtering out low-quality and manually edited sequences, 6,676 nearly full-length sequences remained. Among these, HRSV A accounted for 3,449 sequences, while HRSV B comprised 3,227. Specifically, prior to 2008, particularly before 2001, the number of available sequences was limited, almost exclusively to those from North America. This scarcity was due to less developed sequencing capabilities and lower financial investment. There has been a significant increase in the number of sequences each year since then, especially since 2016. Global sequences from 2008 to 2023 reveal that HRSV A predominated during the periods of 2008 to 2013 and 2022, while HRSV B was more prevalent in 2014 to 2018 and 2023. Notably, in 2019 and 2021, the number of sequences for both subtypes was comparable (Fig. 1). Geographically, during this period, sequences originated from all major continents, with the majority coming from North America and Europe. Africa, more specifically Kenya, also contributed a substantial number of sequences between 2010 and 2019. Notably, circulating patterns of HRSV subtypes varied across continents and time periods. For example, in 2017, HRSV B dominated in Africa while HRSV A was predominant in South America. In 2022, North America was predominantly associated with HRSV A, whereas Europe was dominated by HRSV B. In contrast, no obvious differences in the prevalence of HRSV A and HRSV B were observed across continents in 2019 (Fig. 1). The nearly full-length sequences analyzed in this study can be found in the supplementary file, Supplementary Material online.

### Nucleotide and Amino Acid Entropy in HRSV A and HRSV B

**Fig. 2.**
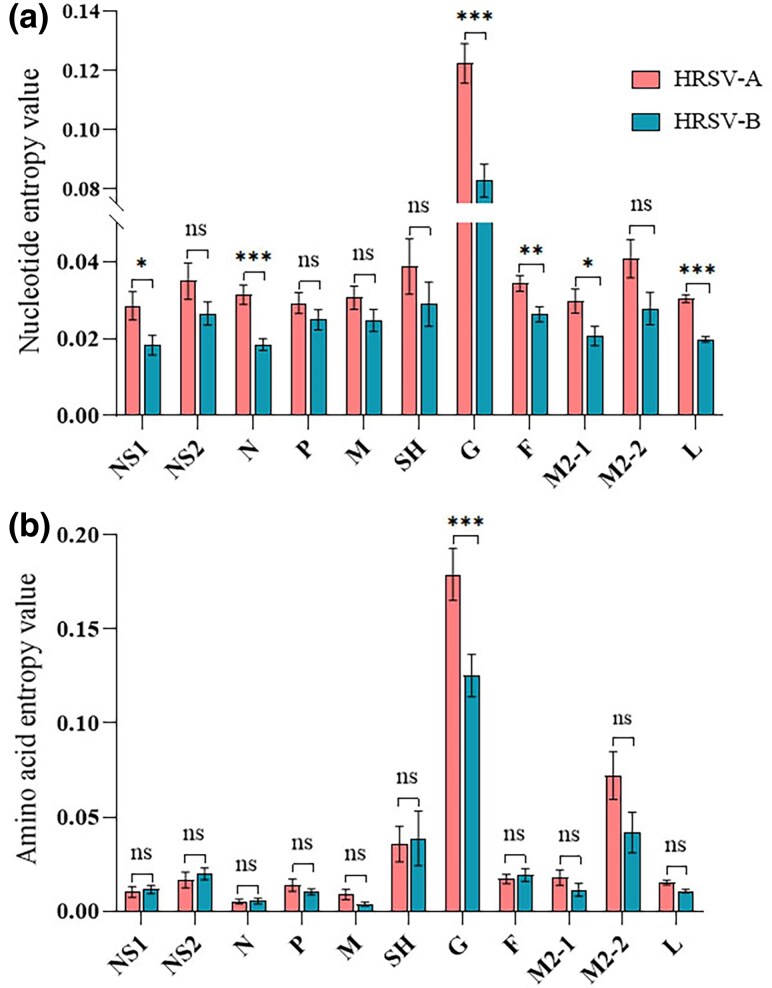
Entropy values for HRSV at nucleotide and amino acid level. a) nucleotide entropy value. The diversities of NS1, N, G, F, M2-1 and L in HRSV A were significantly greater than those in HRSV B. b) Amino acid sequences. Only the G exhibited significantly higher diversity in HRSV A compared to HRSV B. *, *P* < 0.05; **, *P* < 0.01; ***, *P* < 0.001.

We conducted a genome-wide analysis of nucleotide and amino acid entropy values for HRSV A and HRSV B. The results showed that the G presented the highest diversity at both the nucleotide and amino acid levels. Specifically, at the nucleotide level, the entropy values for genes other than G were relatively similar, generally ranging from 0.02 to 0.04. HRSV A exhibited higher entropy values across all genes compared to HRSV B. Further analysis revealed no statistically significant differences in entropy values for the NS2, P, M, SH, and M2-2 genes. In contrast, statistical differences were observed for the NS1, N, G, F, M2-1, and L genes (*P* < 0.05 in one-way ANOVA with post hoc Turkey multiple-comparison test) (Fig. 2a). At the amino acid level, aside from G, the SH and M2-2 proteins also exhibited relatively high entropy values. One-way ANOVA analysis demonstrated that only the G had a significantly higher entropy value in HRSV A compared to HRSV B, while no significant differences were observed for the other proteins between the two subtypes. Interestingly, in contrast to the nucleotide level, where HRSV B generally displayed lower entropy values than HRSV A, the amino acid entropy values for NS1, NS2, SH, and F in HRSV B were slightly higher than those in HRSV A (Fig. 2b).

### SNP Calling Analysis

#### Mutation Rates and Types

**Fig. 3.**
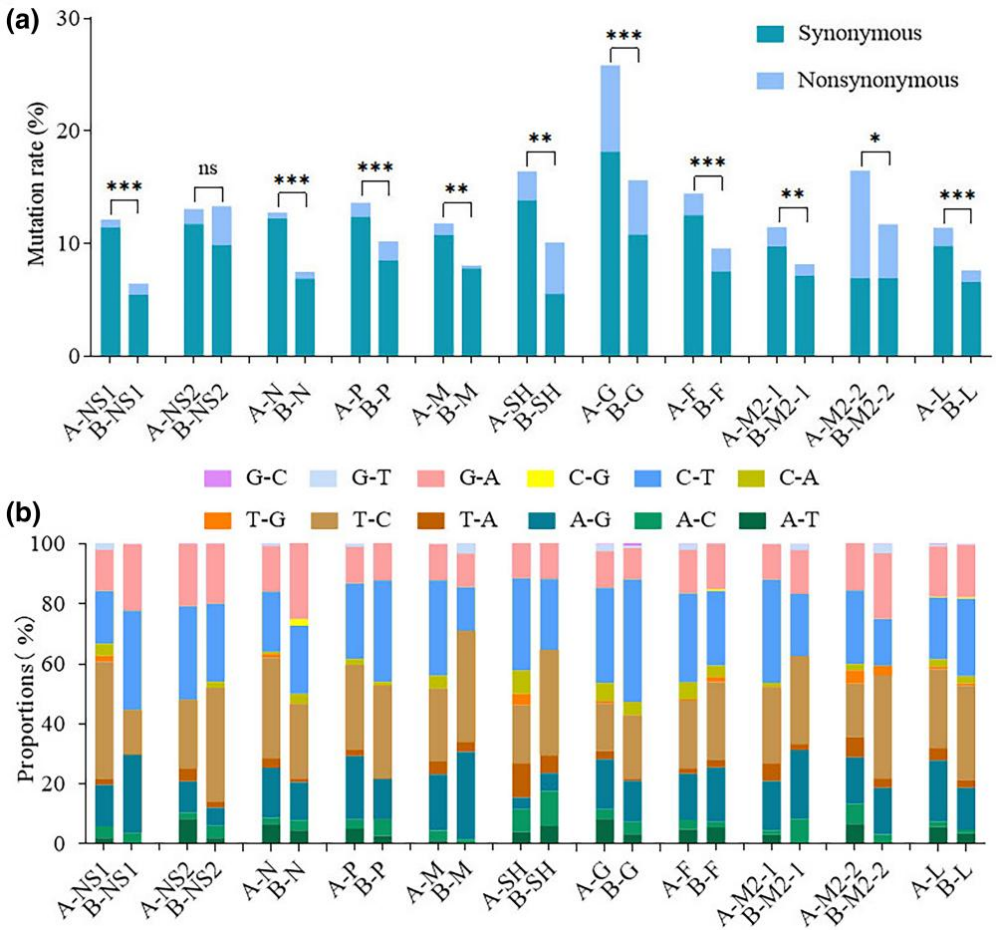
Mutations in each gene of HRSV. a) mutation rate and mutation type. Except for the NS2 gene, the mutation rates of all other genes were significantly higher in HRSV A compared to HRSV B. Synonymous mutations predominated across all genes. *, *P* < 0.05; **, *P* < 0.01; ***, *P* < 0.001. b) mutation directions. C to T, T to C, G to A, and A to G were the most common mutation directions.

We performed SNP calling on the full-length sequences of HRSV A and HRSV B, followed by a detailed assessment of the mutation types for each gene. The results indicated that, except for the NS2 gene, where the mutation rates were comparable between HRSV A and HRSV B, all other genes exhibited higher mutation rates in HRSV A than in HRSV B. Chi-square test analysis revealed no significant difference in the mutation rate of the NS2 gene between the two subtypes; however, statistical differences were observed for all other genes, with HRSV A exhibiting a markedly higher mutation rate than HRSV B. Furthermore, we found that the majority of SNPs in each gene resulted in synonymous mutations. In comparison to other genes, the SH, G, and M2-2 genes of both HRSV A and HRSV B, as well as the NS2 gene of HRSV B, exhibited a higher frequency of nonsynonymous mutations (Fig. 3a). This observation was consistent with the above entropy values, where an increased proportion of nonsynonymous mutations correlated with higher entropy value.

In addition, we conducted a further analysis of the mutation directions in each gene and found that transitions predominated, with C to T, T to C, G to A, and A to G being the most common mutation types. Overall, there were no significant differences in these mutation directions between HRSV A and HRSV B. However, for specific genes such as NS1, HRSV A primarily exhibited T to C transitions, while HRSV B showed a predominance of C to T transitions (Fig. 3b).

#### Codon Positions of SNPs

**Fig. 4.**
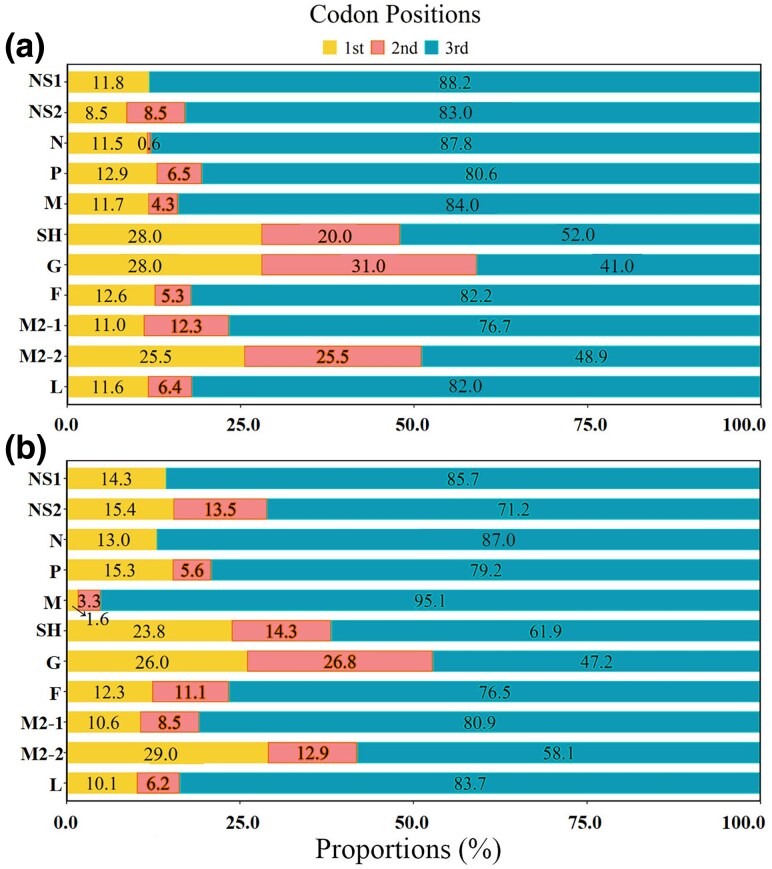
Codon positions for SNPs. a) HRSV A; b) HRSV B. The majority of SNPs were located at the third codon position on each gene.

We analyzed the codon positions of SNPs in each gene of HRSV A and HRSV B and found that the majority of SNPs were located at the third codon position. Specifically, with the exception of the G, SH, and M2-2 genes, over 70% of SNPs in the other genes of HRSV A and HRSV B were found at this position. Notably, 88.2% of SNPs in the NS1 gene of HRSV A and 95.1% in the M gene of HRSV B were located at the third codon position. In contrast, the G gene had the lowest proportion of mutations at the third codon position, with 41.1% for HRSV A and 47.2% for HRSV B. The M2-2 and SH genes also presented relatively low proportions, with SNPs at the third codon position accounting for 48.9% and 58.1% in HRSV A and 52.0% and 61.9% in HRSV B, respectively (Fig. 4).

### Selective Pressures on the Proteins of HRSV A and HRSV B

We conducted selective pressure analyses on each protein of HRSV A and HRSV B. The results showed no positively selected sites in the NS1, NS2, and N proteins of HRSV A, nor in the NS1 and N of HRSV B. However, we identified varying numbers of positively selected sites in the remaining proteins. Notably, the G protein exhibited the highest selective pressure, with 24 positively selected sites in HRSV A and 34 in HRSV B. Among these, 16 sites for HRSV A and 21 for HRSV B were classified as exhibiting strong positive selection. Further analysis revealed that these positively selected sites were predominantly located in Mucin-like region II, which contained 13 sites for HRSV A and 21 for HRSV B, respectively, followed by Mucin-like region I, with 8 sites for HRSV A and 6 sites for HRSV B. The two Mucin-like regions are heavily glycosylated and play critical roles in immune evasion (McLellan et al. 2013), indicating the importance of the regions in the ongoing evolutionary arms race between the virus and the host. In contrast to the highly variable G protein, the F protein, another neutralizing antigen, showed relative stability, with only five and four positively selected sites in HRSV A and HRSV B, respectively, none of which were located in known major neutralizing epitopes. Additionally, we observed a higher number of positively selected sites in the L and M2-2 proteins of both HRSV A and HRSV B, with 10 and 7 positively selected sites in L, and 6 and 7 sites in M2-2 for HRSV A and HRSV B, respectively (Table 1).

**Table 1:** Positively selected sites in HRSV A and HRSV B

Genes	HRSV A	HRSV B
**NS1**	…	…
**NS2**	…	5, **6**, **53**
**N**	…	…
**P**	**66**, 73, **225**	60, 77
**M**	**43**	157
**SH**	38, **55**	**49**, **64**
**G**	94, **101**, 103, **115**, 132, **134**, 151, 161, **198**, 208, 212, 246, **251**, **257**, **264**, **265**, **275**, **276**, **286**, **288**, **299**, **300**, **310**, **312**	**4**, 6, **19**, 21, 98, **135**, **137**, 143, 151, **154**, 195, **215**, **219**, **231**, 235, **251**, **255**, **258**, **260**, **262**, **271**, **274**, **279**, 280, 281, **282**, 285, 287, **291**, 297, 300, **304**, **309**, **316**
**F**	23, **122**, 518, 553, 554	**4**, **5**, **12**, **125**
**M2-1**	**117**, **194**	**121**, **188**
**M2-2**	**1**, **2**, 26, 36, 37, **46**	**26**, **27**, **35**, 42, 48, **49**, **79**
**L**	143, **216**, 943, 1657, 1724, 1725, 1804, 1943, 2113, **2163**	**166**, **1718**, **1750**, 1759, 2111, 2162

Notes: The “…” indicates the gene has no positively selected sites; bolded sites are positively selected in all the three methods (MEME, FUBAR, and SLAC), while underscored sites are positively selected in two of the three methods.

### Major Lineages of HRSV Circulating During and After the COVID-19 Pandemic

**Fig. 5.**
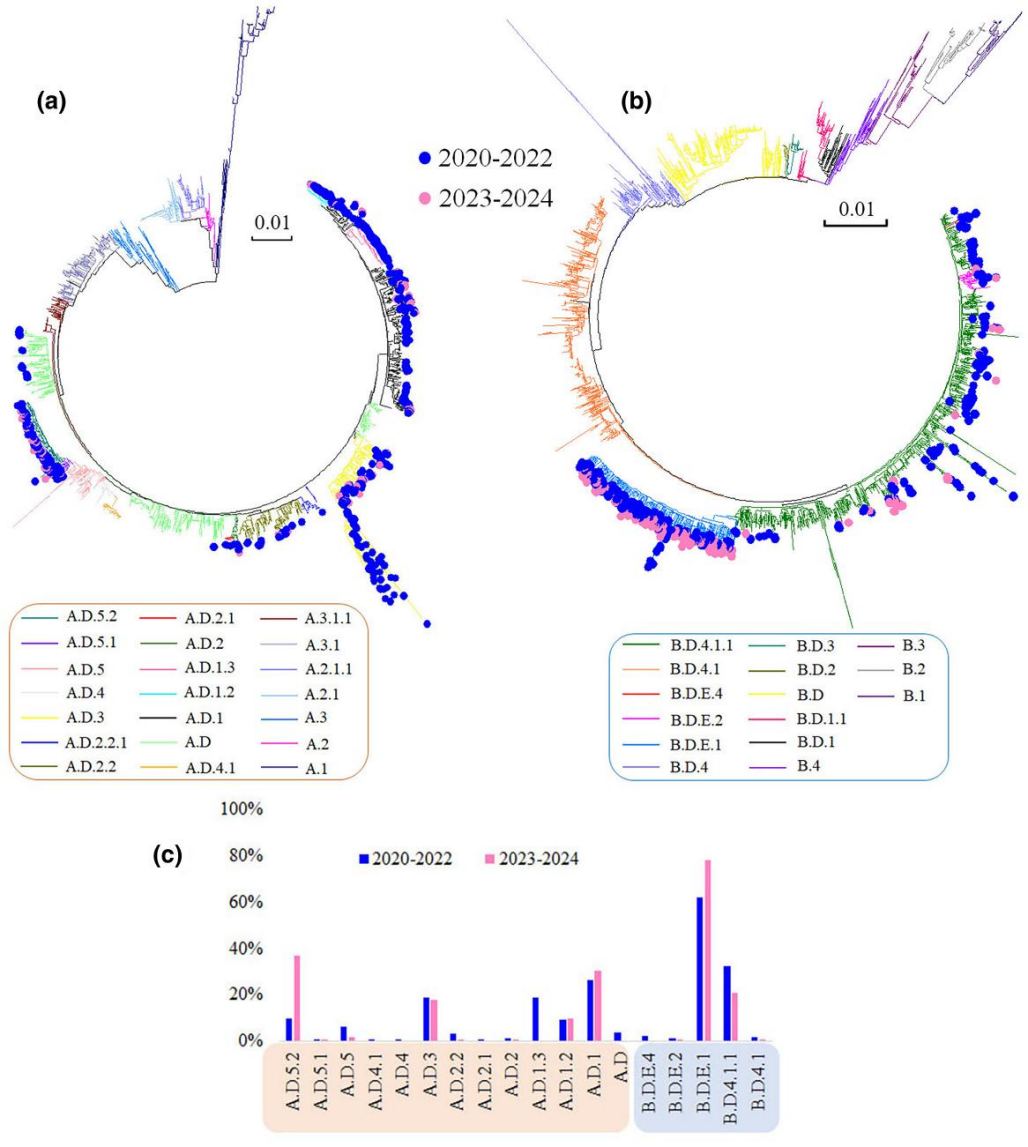
Lineages of HRSV A and HRSV B over the last 5 years. a) HRSV A. The sequence distribution during the pandemic comprised 13 lineages, whereas post-pandemic has significantly decreased. b) HRSV B. The sequence distribution prior to the pandemic included 5 lineages, with two being predominant. Post-pandemic, the distribution became restricted to these two main lineages. c) Proportional distribution of lineages for sequences over the past 5 years. The predominant circulating lineages of HRSV A have changed before and after the pandemic, while those of HRSV B have remained relatively stable.

We constructed phylogenetic trees using the full-length sequences of both HRSV A and HRSV B. The analysis revealed that the sequences included in this study encompassed 21 lineages for HRSV A and 15 lineages for HRSV B, as defined by the latest Nextstrain nomenclature for HRSV evolutionary lineages (Fig. 5a and b). Further investigation of HRSV A sequences over the past 5 years highlighted significant lineage diversity during the pandemic period (2020 to 2022), with sequences distributed across 13 different lineages. The predominant lineages were A.D.1, A.D.1.3, and A.D.3, collectively accounting for 64.2% of all sequences, with individual contributions of 26.2%, 19.1%, and 18.9%, respectively. Other noteworthy lineages included A.D.5.2, A.D.1.2, and A.D.5, which represented 9.9%, 9.4%, and 6.1% of the total sequences, respectively. In contrast, the diversity of HRSV A sequences decreased after the pandemic (2023 to 2024), with only 8 different lineages identified. During this period, the dominant lineages were A.D.5.2, A.D.1, A.D.3, and A.D.1.2, which together accounted for 95.5% of the total, with individual proportions of 36.9%, 30.6%, 18.1%, and 9.9% (Fig. 5c). The remaining lineages were represented by fewer than five sequences. Analysis of HRSV B sequences over the same 5-year period showed fewer circulating lineages compared to HRSV A, with only 5 versus 13 lineages identified. The primary lineages throughout this period were B.D.E.1 and B.D.4.1.1, accounting for a combined proportion of 64.6% before the pandemic and 99.1% after. Lineages B.D.E.2, B.D.E.4, and B.D.4.1 were intermittently detected during the pandemic but were not observed after its conclusion (Fig. 5c).

### Temporal Distribution of Lineage-Defining Amino Acids on Major Circulating Strains Post-Pandemic

**Fig. 6.**
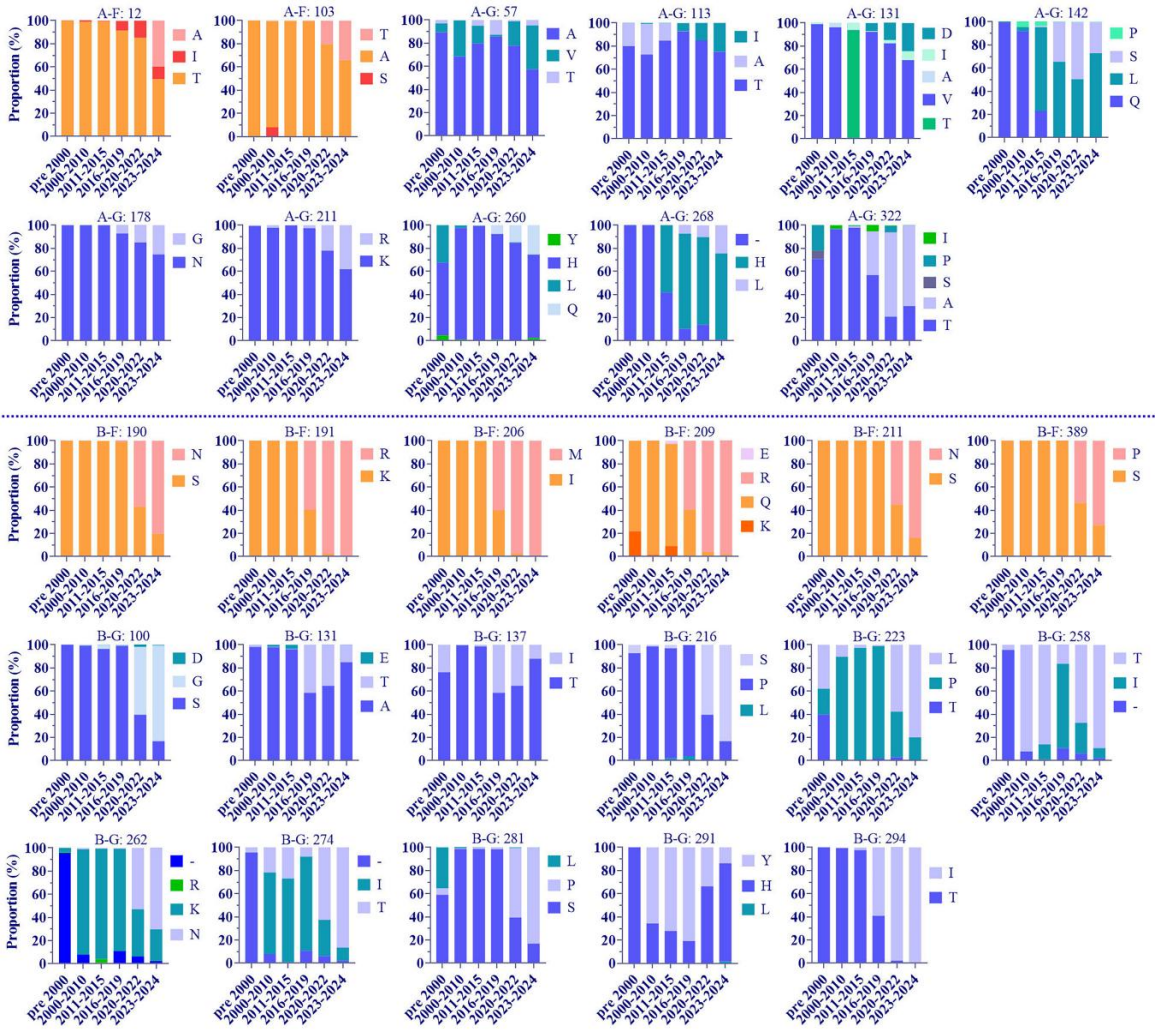
Temporal distribution of substitutions on lineage-defining amino acids in F and G proteins of major circulating strains post-pandemic. The substitutions in the F protein of HRSV A and HRSV B exhibited temporal specificity, while only part of the substitutions in the G protein showed temporal specificity.

To understand the antigenic characteristics of dominant lineages post-pandemic, we analyzed lineage-defining amino acid changes and their temporal distribution on the neutralizing antigens F and G (Goya et al. 2024). For HRSV A, we examined the following substitutions: T12I and A103T in F protein, A57V, L142S, T113I, V131D, N178G, K211R, H260Q, 268H-L, and T322A in G protein. The results indicated that position 12 harbored both A and I amino acid changes, with A being a specific substitution that emerged after the pandemic, while A103T was present during and after the pandemic. The substitutions T113I, V131D, N178G, K211R, H260Q, and H268L showed an increasing proportion over time, whereas A57V, L142S, and T322A did not demonstrate a clear temporal pattern (Fig. 6).

For HRSV B, we examined the following substitutions: S190N, K191R, I206M, Q209R, S211N, S389P in F protein and A131T, T137I, S100G, P216S, P223L, I258T, K262N, I274T, S281P, Y291H, and T294I in G protein. Among the six lineage-defining amino acid substitutions in the F protein, four were located at major neutralizing epitopes: three (I206M, Q209R, and S211N) at site ∅ and one (S389P) at site I. The remaining two (S190N and K191R) were very adjacent to site V. Importantly, substitutions S190N, S211N, and S389P emerged during the pandemic, with frequencies exceeding 60% post-pandemic. In contrast, K191R, I206M, and Q209R surpassed 50% during the 2016 to 2019 period, reaching nearly 100% prevalence during and after the pandemic. Among the eleven lineage-defining amino acid substitutions in the G protein, positions 137, 258, 262, 274, and 291 were under strong positive selection (Table 1). Substitutions S100G, P216S, K262N, and S281P primarily occurred during the pandemic and post-pandemic, with predominance after the pandemic. Conversely, A131T and T137I had the highest proportions during the 2016 to 2019 period, showing a decline in prevalence during and after the pandemic. Additionally, substitutions P223L, I258T, I274T, and T294I were present before the pandemic, with significantly increased proportions during and after the pandemic (Fig. 6).

Furthermore, most lineage-defining amino acids in the G protein of both HRSV A and HRSV B were located in the highly variable mucin-like regions I and II, while A57V in HRSV A was situated in the transmembrane region (TM), and K211R in HRSV A, P216S, and P223L in HRSV B were located in the heparin-binding domain (HBD). Notably, N178G in HRSV A was positioned in the central conserved domain (CCD).

## Discussion

Since its first discovery in 1955 (Blount et al. 1956; Chanock et al. 1957), HRSV has been circulating for nearly 70 yr. A comprehensive understanding of its epidemiological pattern and transmission patterns, as well as the implementation of effective control measures, relies on in-depth research into its genomic features. In this study, we analyzed the evolutionary and variation characteristics of HRSV using whole-genome sequences from public databases. The data distribution indicated a significant increase in HRSV genome sequencing since 2010, particularly after 2015. We observed that HRSV A and HRSV B alternate in dominance every few years, and this pattern exhibits geographical variation. This geographic heterogeneity has been previously documented. In China, the dominant subtype pattern from 2008 to 2021 was ABBAABAABAAABB (Song et al. 2023), while in the United States, excluding the 1-year missing data for 2020 to 2021, the predominant subtype pattern over the past 13 years has been ABAABBABBAABA (Rios-Guzman et al. 2024). These regional differences in subtype prevalence likely result from varying levels of population immunity to different subtypes, as well as potential environmental or socioeconomic influences that may favor the spread of one subtype over another. However, the current sequencing data are primarily concentrated in a limited number of countries/regions, leaving many others with relatively sparse data. To better understand the epidemiology and variation characteristics of RSV and mitigate the risks associated with gaps in monitoring, it is essential to enhance global genome sequencing efforts, which will promote international scientific cooperation and data sharing, facilitating the development of targeted RSV control strategies.

Our results indicated that the genetic diversity of HRSV A was generally higher than that of HRSV B. With the exception of the NS2 gene, mutation rates for all other genes were significantly higher in HRSV A compared to HRSV B. This difference might stem from the NS2 protein performing key functions such as inhibiting the host's interferon response in both subtypes, and its interaction interface with specific host factors, like interferon pathway proteins, is highly conserved, thus limiting inter-subtype differences in mutation rates. This also highlights the functional stratification strategy of different HRSV genes in host adaptation. Consequently, the nucleotide entropy values for the genes in HRSV A were consistently higher than those in HRSV B, with statistically significant differences observed in six genes. However, only the amino acid entropy value of the G protein in HRSV A was significantly higher than in HRSV B. Moreover, although SNPs for all genes primarily occurred at the third position of the codon, the proportions of SNPs at the first and second positions in the G, SH, and M2-2 genes were much higher. Previous studies have shown that due to codon degeneracy, mutations at the third position of the codon were more likely to be synonymous than those that occurred at the first or second positions (Bofkin and Goldman 2006). We also observed a relatively high proportion of nonsynonymous mutations in these three genes. Additionally, while it is well known that APOBEC-mediated C-to-T transitions account for nearly half of the mutation types in the SARS-CoV-2 genome (Sanderson et al. 2023), we found that four types of transitions (C to T, T to C, G to A, and A to G) were commonly present in all the genes of HRSV. The difference between the two viruses may be attributable to varying selective pressures exerted by the host, distinct replication dynamics, or asymmetric interactions with host RNA-editing enzymes specific to each subtype in the NS1 gene. Further investigation is warranted to explore the underlying biological mechanisms.

Evolutionary information has been utilized to enhance insight into protein function and structure. Positive selection, or diversifying selection, refers to the process by which natural selection favors nonsynonymous mutations that may confer a survival or reproductive advantage to individuals (Daugherty and Malik 2012). In our study, we found that the G proteins of both HRSV A and HRSV B exhibited a significant number of positively selected sites, consistent with previous studies (Botosso et al. 2009; Kim et al. 2023). Additionally, we detected several sites under strong positive selection in the F, M2-2, and L proteins. The G and F proteins are well-known neutralizing antigens for HRSV, and their positively selected sites may be related to viral attachment, infection, and immune evasion. Compared to our earlier studies (Yu et al. 2021; Guo et al. 2023), the number of sites under positive selection in the F and G proteins of HRSV A and HRSV B has markedly increased. Furthermore, this study identified sites under positive selection in SH, M, P, and M2-1 proteins. The presence of these positively selected sites, particularly in the polymerase L protein, the replication-associated factor P and M2-1, and the M2-2 protein, involved in switching between transcription and replication, may influence the viral replication capability (Musa et al. 2024).

Air travel plays a significant role in the global spread of influenza viruses and SARS-CoV-2 (Lemey et al. 2014; Bedford et al. 2015; Lemey et al. 2021), and it also serves as a predictor for the global dispersal of HRSV (Langedijk et al. 2024). A recent study has shown that the COVID-19 pandemic interventions have altered the global transmission patterns of seasonal influenza viruses (Chen et al. 2024). Similarly, implementation of public health and social measures has resulted in a dramatic reduction in HRSV activity, alongside a shift in seasonality and a delayed outbreak with a great number of infected patients in numerous countries (Chuang et al. 2023). Additionally, there have been notable changes in the circulating lineages of HRSV A and HRSV B post-pandemic, with HRSV A primarily consisting of the A.D.5.2 and A.D.3, while HRSV B was predominantly composed of the B.D.4.1.1 and B.D.E.1 (Cho et al. 2024; Wei et al. 2024). Our study also showed that A.D.5.2 and B.D.E.1 were the most prevalent circulating strains of HRSV A and HRSV B post-pandemic. However, over 60% of HRSV A consists of three other lineages—A.D.1, A.D.3, and A.D.1.2—while about 20% of HRSV B is presented by the lineage B.D.4.1.1. This suggested that the dominant strain B.D.E.1 may have a greater adaptability within the population, or its prevalence could be the result of random genetic drift after bottlenecks in the viral population. Further research is needed to shed light on this phenomenon. In contrast, the coexistence of multiple lineages in HRSV A may reflect a faster evolutionary rate, and its greater diversity potentially facilitates its sustained transmission within the population. Furthermore, we observed a higher number of circulating lineages of HRSV A and HRSV B during the pandemic compared to the post-pandemic period. We speculated that some of the lineages, such as A.D.1.3, may have gone extinct due to reduced transmission caused by viral population bottlenecks, which were likely the result of non-pharmacological interventions implemented to mitigate SARS-CoV-2 transmission. However, given the limited number of sequences available from the post-pandemic period, we cannot rule out the possibility that sampling and sequencing biases have resulted in the undetected circulation of these lineages in other regions. Therefore, ongoing and larger-scale sequencing efforts are essential for comprehensive HRSV surveillance.

The major prevalent lineages of HRSV post-pandemic were characterized by substitutions in lineage-defining amino acids across multiple proteins (Goya et al. 2024). For example, lineage A.D.5.2 exhibited a T89I in the P protein, lineage A.D.3 presented a T79A in the M2-2 protein, lineage B.D.E.1 had an R1759K in the L protein, and lineage B.4.1.1 featured an N64D in the SH protein. The F and G proteins served as the primary neutralizing antigens of HRSV, playing crucial roles in membrane fusion and receptor binding during the viral infection. In the currently circulating lineages, the F protein of HRSV A contained two lineage-defining amino acid sites, while HRSV B had six. Notably, the two sites in HRSV A were located away from the neutralizing antigenic epitopes, whereas four of the sites in HRSV B were situated on the major neutralizing antigenic epitopes, with the remaining two located very close to the epitopes. *In vitro* studies demonstrated similar neutralization efficacy of nirsevimab against HRSV A and HRSV B (IC50 values of 3.1 and 3.0, respectively) (Zhu et al. 2017), certain HRSV B variants may exhibit reduced susceptibility. Specifically, the I206M/Q209R double mutation in HRSV B has been shown to moderately increase susceptibility to nirsevimab (Zhu et al. 2018). Furthermore, in the Phase III trial of nirsevimab in preterm infants, two clinical HRSV B isolates from nirsevimab recipients presented resistance-associated substitutions (Griffin et al. 2020). Moreover, the single substitution K272E has been identified as causing palivizumab (PZ) escape or resistance in both animal models and patients (Oliveira et al. 2015; Jo et al. 2021). Currently, two preF protein-based HRSV subunit vaccines are on the market: Arexvy, which is a monovalent vaccine, and ABRYSVO, which is a bivalent vaccine targeting both A and B subtypes. As of now, there are no data available to indicate whether the mutations on the F protein post-epidemic impact the efficacy of these vaccines. Therefore, continuous monitoring of substitutions, including S211N, is crucial to understand their potential impact on viral characteristics.

Regarding the G protein, HRSV A and HRSV B contained 9 and 11 lineage-defining amino acid sites, respectively. Interestingly, five of these sites in HRSV B were under strong positive selection, while none of the sites in HRSV A experienced significant selective pressure. Previous studies have demonstrated that, compared to HRSV A, both the complete genomic and G gene evolutionary rates of HRSV B were slightly higher (Martinelli et al. 2014; Pangesti et al. 2018; Yu et al. 2021). Additionally, HRSV B exhibited a greater number and frequency of amino acid changes at antigenic sites of the F protein than HRSV A (Hause et al. 2017). Thus, considering that HRSV B exhibited a greater number of unique substitutions in both F and G, with several occurring at key neutralizing antigenic sites on F in this study, we speculated that HRSV B may evade host immune surveillance more effectively during infection, and the evolutionary strategies of HRSV A and HRSV B might be distinct: HRSV B may achieve immune evasion through “antigenic site-directed evolution”, while HRSV A might rely on the “accumulation of multi-site variations” to sustain its transmission.

It is noteworthy that, while most lineage-defining amino acids of the G protein were located in hypervariable regions, an amino acid substitution from N to G occurs at position 178 in the CCD of HRSV A. The CCD is devoid of glycosylation and contains a CX3C motif that facilitates binding to the receptor CX3CR1, playing key roles in virus infection and viral pathogenesis (Johnson et al. 2015). It has been reported that the insertion of G into the CX3C motif (resulting in CX4C) inhibited receptor binding and significantly reduced disease severity in vivo (Boyoglu-Barnum et al. 2017). Additionally, the S177Q maintained strong binding to protective monoclonal antibodies and exhibits comparable reactivity to human reference antibodies as the wild-type RSV G protein (Nunez Castrejon et al. 2022). The functional implications of the N178G remain unclear and warrant further investigation.

Multiple amino acid substitutions, including A103T and S211N in the F protein, as well as K211R and K262N in the G protein, have emerged during the pandemic and have significantly increased in prevalence post-pandemic. This indicated that these changes were closely associated with viral adaptation and immune evasion. Additionally, we have observed a transition in the F protein of HRSV A from T12I to T12A post-pandemic, the significance of which requires further investigation. Importantly, akin to the critical role of antibody Fc-mediated effector functions in combating SARS-CoV-2 (Zhang et al. 2023), RSV-specific Fc qualities and IgG/IgA Fc-effector functions played a crucial role in enhancing protection against RSV, surpassing the importance of neutralization alone (Bartsch et al. 2022). Therefore, it is essential to pay close attention to mutations outside of neutralizing antigens in the circulating strains of HRSV. Furthermore, mutations in replication-related proteins, such as M2-1 and L, may potentially influence the viral biological properties, underscoring the need for further analysis.

In summary, our study elucidated the evolutionary dynamics and genetic diversity of HRSV, especially during and after pandemic, underscored the critical need for continuous investigation into mutations in both neutralizing antigens and other proteins. By deepening our understanding of HRSV epidemiology and evolution, the study provided essential insights to inform the development of targeted control strategies and interventions aimed at mitigating the impact of HRSV on public health. Future studies will prioritize comprehensive genomic surveillance to track the emergence of new variants and evaluate their potential effects on vaccine efficacy and antiviral treatments, ultimately strengthening public health responses.

## Materials and Methods

### Data Retrieval and Selection

Along with the 3,886 nearly full-length sequences downloaded in our previous study (Williams et al. 2021), we have also collected the nearly full length (≥14,500 nt) of HRSV sequences updated from the GenBank database between January 1, 2023 and May 31, 2024. Sequences manually modified in the laboratory have been excluded from this study.

### Sequence Processing and Annotation

The raw data of nearly full-length sequences were aligned by the MAFFT 7 online version 7 and then manually refined with MEGA 7 software (version 7.0.26). Each sequence was annotated with relevant information, including the GenBank number, subtype, sample collection time, and location. Sequences of the 11 genes of HRSV were extracted from the nearly full-length sequences. Any truncated sequences containing low-quality regions—such as gaps, Ns, or insertions and deletions that resulted in frameshift mutations—were excluded from the study. This process was also applied to the full-length sequences.

### SNP Calling

Genetic diversities for both HRSV A and HRSV B were analyzed, and the single-nucleotide polymorphism (SNP) calling was developed. The compiled R codes were deposited in Github (DOI. 10.5281/zenodo.7077349) as previously described (Zhang et al. 2022). In this study, the HRSV A sequence from 1981 (MG642057) and the HRSV B sequence from 1984 (KP258709), both collected in the United States, were used as reference sequences for the SNP analysis.

### Shannon Entropy Calculation

To overview the evolutionary pattern, we calculated the genome-wide nucleotide and amino acid sequence diversity by determining site-by-site Shannon entropy values using the Entropy-One Tool (https://www.hiv.lanl.gov/content/sequence/ENTROPY/entropy_one.html).

### Selective Pressure Analysis

Statistically supported sites with diversifying selection were analyzed using the Hyphy package (version 2.5.62). The Mixed Effects Model of Evolution (MEME)—assuming varying pressure across branches on the phylogenetic tree, fast unbiased Bayesian approximation (FUBAR)—assuming constant pressure on the entire tree, and single-likelihood ancestor counting (SLAC)—inferring ancestral characters for individual site across the phylogeny, were used for the analysis (Murrell et al. 2012; Suchard et al. 2018; Spielman et al. 2019). To reduce the possibility of occasional events, the sites supported by at least two methods were considered as candidates under positive selection, while those supported by all three methods were defined as strongly selected sites (*P*-value of <0.1 in MEME and SLAC, posterior probabilities of >0.9 in FUBAR).

### Phylogeny Analysis and Temporal Distribution

Phylogenetic analysis was performed on the aligned complete genome sequences of both HRSV A and HRSV B, after filtering out low-quality sequences. To determine the optimal substitution model for the dataset, we utilized ModelFinder within IQ-TREE software (v2.4.0), which identified the General Time Reversible model with Gamma-distributed rate heterogeneity and a proportion of invariant sites (GTR + I + G) as the best-fit model for both HRSV A and HRSV B. Subsequently, maximum likelihood phylogenetic trees were constructed using software of MEGA 12, implementing the selected GTR model with gamma-distributed rates and invariant sites (G + I). The sequences collected after 2020 were divided into two time periods: 2020 to 2022 and 2023 to 2024, and each was then labeled accordingly. After extracting the temporal data of lineage-defining amino acids for the circulating strains post-pandemic, the results were plotted using GraphPad Prism (version 8.0.2.263).

## Supplementary Material

evaf093_Supplementary_Data

## Data Availability

No new data were generated or analyzed in support of this research.
